# Supplementary material for: Integumentary structure and composition in an exceptionally well-preserved hadrosaur (Dinosauria: Ornithischia)
Source: PeerJ. 2019 Oct 16;7:e7875. doi: 10.7717/peerj.7875 (PMC6800526; doi:10.7717/peerj.7875)
Supplement: Supplemental Information 1 [file peerj-07-7875-s001.pdf]

Integumentary structure and composition in an exceptionally well-preserved hadrosaur (Dinosauria: Ornithischia): Supplemental Material

Mauricio Barbi[1], Phil R. Bell[2], Federico Fanti[3,4], James J. Dynes[5], Anezka Kolaceke[1], Josef Buttigieg[6], Ian M. Coulson[7], and Philip J. Currie[8]

[1]**Department of Physics, University of Regina, Regina, Saskatchewan, Canada**
[2]**School of Environmental and Rural Science, University of New England, Armidale, New South Wales, Australia**
[3]**Museo Geologico Giovanni Capellini, Università di Bologna, Bologna, Italy**
[4]**Dipartimento di Scienze Biologiche, Geologiche e Ambientali, Alma Mater Studiorum, Università di Bologna, Bologna, Italy**
[5]**Canadian Light Source Inc., University of Saskatchewan, Saskatoon, Saskatchewan, Canada**
[6]**Department of Biology, University of Regina, Regina, Saskatchewan, Canada**
[7]**Department of Geology, University of Regina, Regina, Saskatchewan, Canada**
[8]**Biological Sciences, University of Alberta, Edmonton, Alberta, Canada**

Corresponding author:
Mauricio Barbi[1]

Email address: barbi@uregina.ca

ABSTRACT

Additional figures and information are included in this supplemental material. XRF data are provided showing iron and copper fingerprinting the skin. The SM spectra from points 1, 3, 4, 5 and 6 identified in Fig. 2C, the full mid-infrared spectra collected from the areas depicted in Fig. 6 and from the "light-coloured powder" sample, and the K-edge analyses of Al, Mg and Si are also included.

1 XRF CHEMICAL MAPPING

X-ray Fluorescence maps (Fig. S1 for the hadrosaur skin sample were generated at the VESPERS beamline station at the Canadian Light Source (Feng et al., 2007). VESPERS is a bending magnet beamline, with energy ranging between 6 and 30 keV. The polychromatic beam used in this measurement is focused using KB mirrors to a beam spot of 2-4 μm by 2-4 μm, adjusted with the help of slits.

2 SEM SPECTROSCOPY FROM A SAMPLE OF UALVP 53290 SKIN AND ASSOCIATED SEDIMENTS

Six points were selected for SEM chemical analysis from a thin section of hadrosaur skin and the underlying sediments (Fig. 2C). The resultant spectrum from point 2 (which corresponds to the area interpreted as skin [dark region in Fig. 2C]) is described and figured in the main text (Fig. 3). The remaining spectra are described here in order from outermost (superficial) to innermost (deep).

The spectrum from point 6 (Fig. S2) suggests the presence of dolomite ($CaMg(CO_3)_2$), or a combination of magnesium oxide (MgO) and calcium carbonate ($CaCO_3$). The spectrum collected from point 1 (Fig. S6) is related to barite ($BaSO_4$). Barite is present only locally and likely represents authigenic mineralization that occurred during diagenesis of the host sediment. Therefore, it likely represents authigenic mineralization that occurred during diagenesis. The uniform appearance of the brighter area in Fig. 2C,

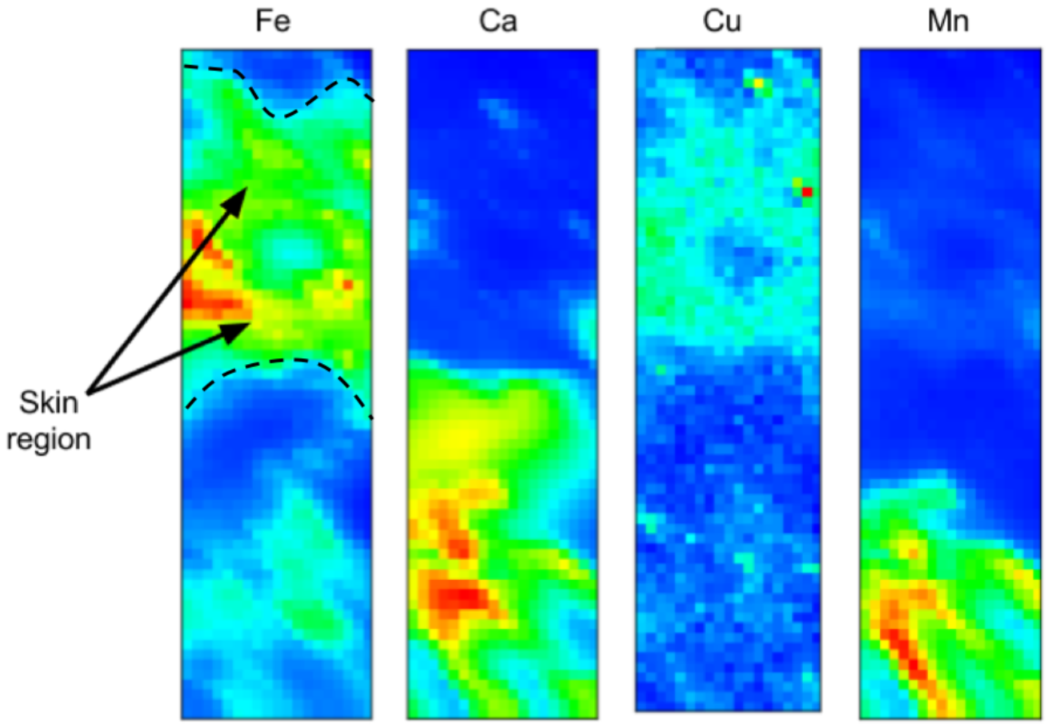

Figure S1. XRF distribution for iron, calcium, copper and manganese. Each pixel measures 25 μm^2; the entire mapped area measures 89.9 μm by 330 μm. Warmer colours correspond to higher concentrations for each element. Maps were plotted using PyMCA (Solé et al., 2007). The concentration of iron, and to a lesser extent, copper, is higher in the region preserving the integument (region between the dashed lines) than to the sediment surrounding it.

where point 1 is located, suggests that this layer is predominantly composed of barite. The spectra from points 5 (Fig. S3) and 4 (Fig. S4) indicate the presence of a clay mineral, kaolinite ($Al_4[Si_4O_{10}](OH)_8$) and an alkali-feldspar ($(NaK)AlSi_3O_8$) (detritus from an igneous rock parent), respectively. Point 3 (Fig. S5) is composed of silica and oxygen, and represents a grain of quartz.

3 FULL SET OF MID-INFRARED SPECTRA

Figures S7 and S8 show the complete set of spectra collected from the skin samples labelled ”dark-coloured powder” and ”light-coloured powder”, respectively, at the MidIR beamline endstation.

4 AL, MG AND SI K-EDGE ANALYSES FROM UALVP 53290 SKIN SAMPLE

4.1 Al K-edge

Aluminum is generally coordinated to either 4 or 6 groups. The Al K-edge spectrum can differentiate between 4-fold and 6-fold coordination environments around the Al. Al compounds with 6-fold coordination have two maxima at 1567.7 $\pm$ 0.3 eV and 1571.5 $\pm$ 0.4 eV, while 4-fold coordinated compounds have a single maximum at 1566.2 *pm* 0.7 eV (Hu et al., 2008).

Fig. S9 shows an Al sequence with combination of spectra representative of 4-fold ($AlPO_4$), 6-fold (gibbsite, $Al(OH)_3$) and 4, 6-fold (muscovite/sericite, $KAl_2(AlSi_3O_{1}0)(F,OH)_2$) coordinated spectra. Threshold masking of the component maps indicated that the spectra best matches that of a 4-fold and 4- and 6-fold coordinated Al (Fig. S9). Discrete particles of both the 4-fold and 4, 6-fold coordinated Al compounds are present in the sample. The 4-fold Al species spectrum is similar to albite ($Na[AlSi_3O_8]$) (likely K-feldspar ($K[AlSi_3O_8]$) given the significant K content in the skin and its presence in the sediment) and the 4, 6-fold Al species is similar to that of muscovite/sericite also present as an alteration product of

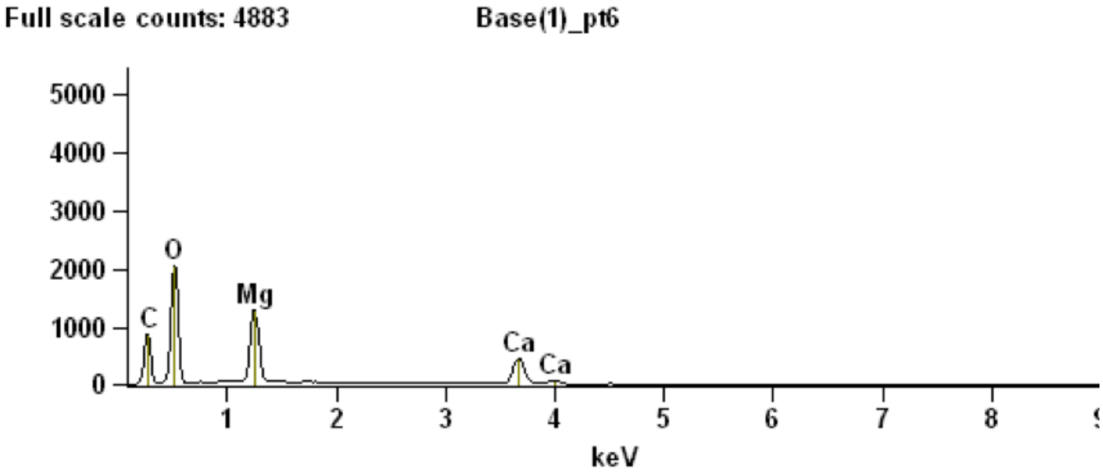

Figure S2. SEM spectrum from point 6 as indicated in Fig. 2C.

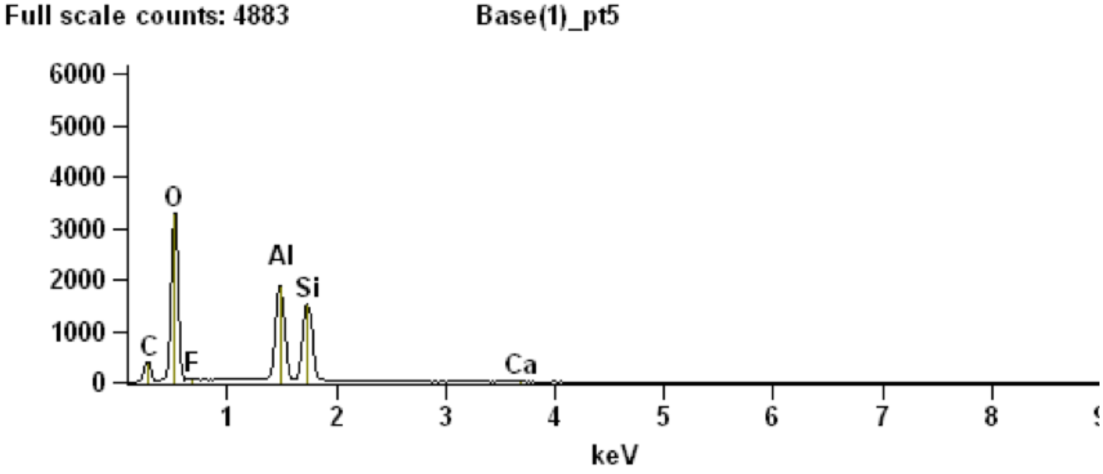

Figure S3. SEM spectrum from point 5 as indicated in Fig. 2C.

albite within the host sediment (Ildefonse et al., 1998). Overlay of the 4-fold component map with the Si species 1 component map confirmed that it is an Aluminum-silicate (data no shown).

4.2 Mg K-edge

Only one Mg species, which occurs as crystal (sediment) in the skin, was apparent from the PCA-CA. The component map derived using this Mg spectrum is shown in Fig. S10. Comparisons between the spectrum obtained from the sample and the reference spectra indicated that the Mg is likely due to $CaCO_3$ associated with significant amounts of MgO and/or dolomite ($CaMg(CO_3)_2$) (Finch and Allison, 2007; Yoshimura et al., 2013). The Mg may also relate to chlorite, which was observed within the host sediment.

4.3 Si K-edge

Two Si species were apparent from the PCA-CA (Fig. S11a). Their Si K-edge spectra along with the component maps derived using these spectra are shown in Figs. S11b,c,d. The Si Species 1 was attributed to be a tectosilicate, likely K-feldspar since it is co-localized with K and the 4-fold coordinated Al (Ildefonse et al., 1998). The main peak of the Si species 2 is about 0.2 eV higher than the main peak for Si species 1. Li et al. (1995) found that the main peak for SiO_2 (e.g., quartz, coesite, cristobalite) is about 0.1 to 0.3 eV higher than albite and K-feldspar. The fact that Si species 2 is not co-localized with the Al component maps (not shown) supports the contention that it is SiO_2.

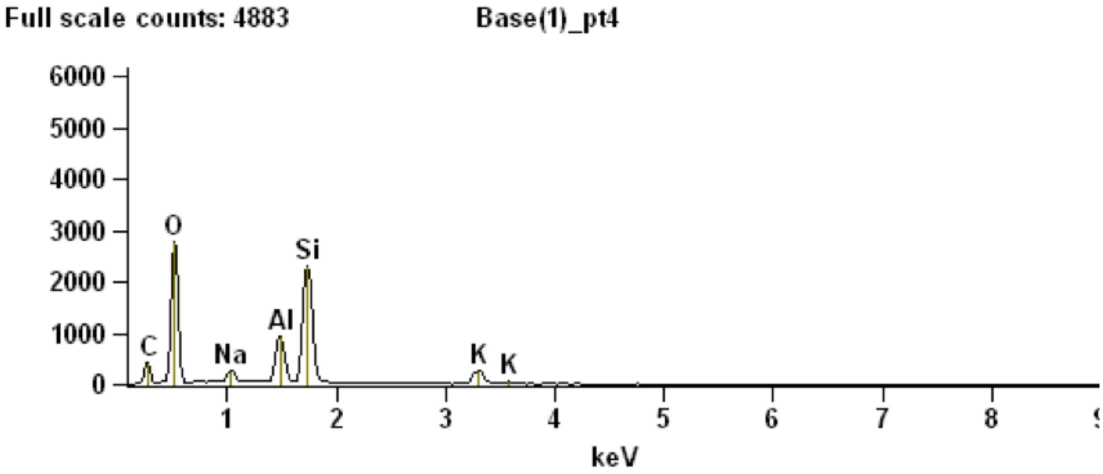

Figure S4. SEM spectrum from point 4 as indicated in Fig. 2C.

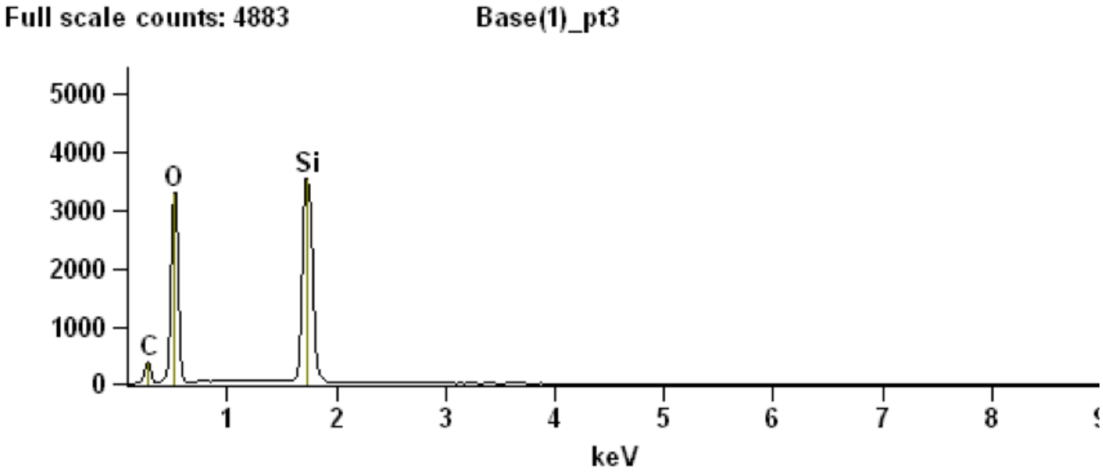

Figure S5. SEM spectrum from point 3 as indicated on Fig. 2C.

REFERENCES

Feng, R., Gerson, A., Ice, G., Reininger, R., Yates, B., and S., M. (2007). Vespers: A beamline for combined xrf and xrd measurements. *AIP Conference Proceedings*, 879(1):872–874.

Finch, A. and Allison, N. (2007). Coordination of sr and mg in calcite and aragonite. *Mineralogical Mag.*, 71:539–552.

Hu, Y., Xu, R. K., Dynes, J. J., Blyth, R. I. R., Yu, G., Kozak, L. M., and Huang, P. M. (2008). Coordination nature of aluminum (oxy)hydroxides formed under the influence of tannic acid studied by x-ray absorption spectroscopy. *Geochimica et Cosmochimica Acta*, 72(8):1959–1969.

Ildefonse, P., Cabaret, D., Sainctavit, P., Calas, G., Flank, A.-M., and Lagarde, P. (1998). Aluminium x-ray absorption near edge structure in model compounds and earth's surface minerals. *Phys Chem Minerals*, 25:112–121.

Li, D., Bancroft, G., Fleet, M., and Feng, X. (1995). Silicon k-edge xanes spectra of silicate minerals. *Phys. Chem. Minerals*, 22:115–122.

Solé, V., Papillon, E., Cotte, M., Walter, P., and Susini, J. (2007). A multiplatform code for the analysis of energy-dispersive x-ray fluorescence spectra. *Spectrochim. Acta Part B*, 62:63–68.

Yoshimura, T., Tamenori, Y., Iwasaki, N., Hasegawa, H., Suzuki, A., and Kawahata, H. (2013). Magnesium k-edge xanes spectroscopy of geological standards. *J. Synchr. Rad.*, 20:734–740.

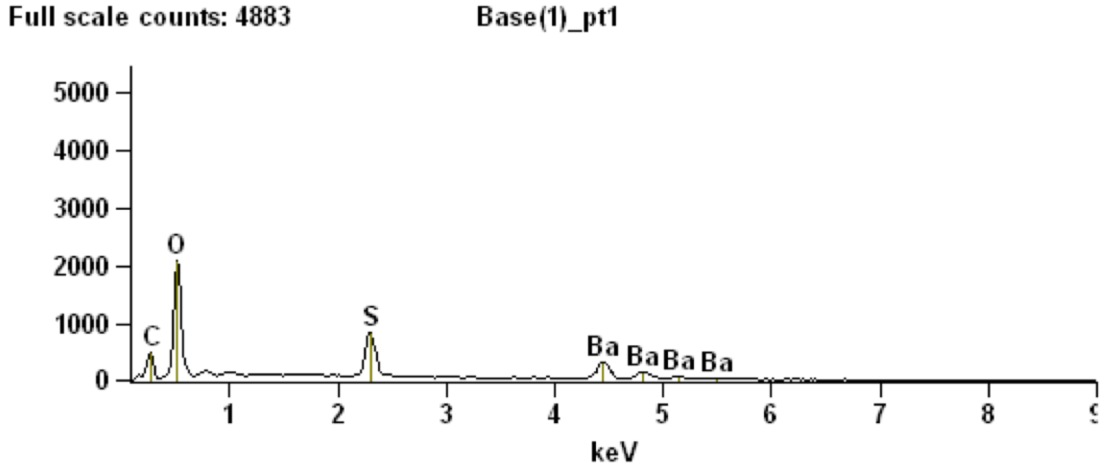

Figure S6. SEM spectrum from point 1 as indicated in Fig. 2C.

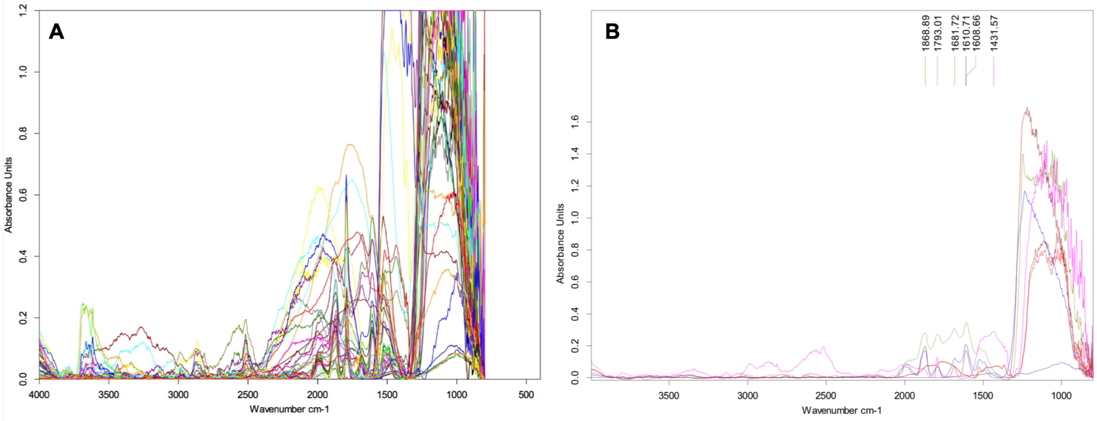

Figure S7. (A) Complete set of spectra collected at the MidIR beamline endstation corresponding to the points indicated in Fig. 7 (each point is represented by a different colour). (B) Set of spectra representative of a line in the map shown in Fig. 7.

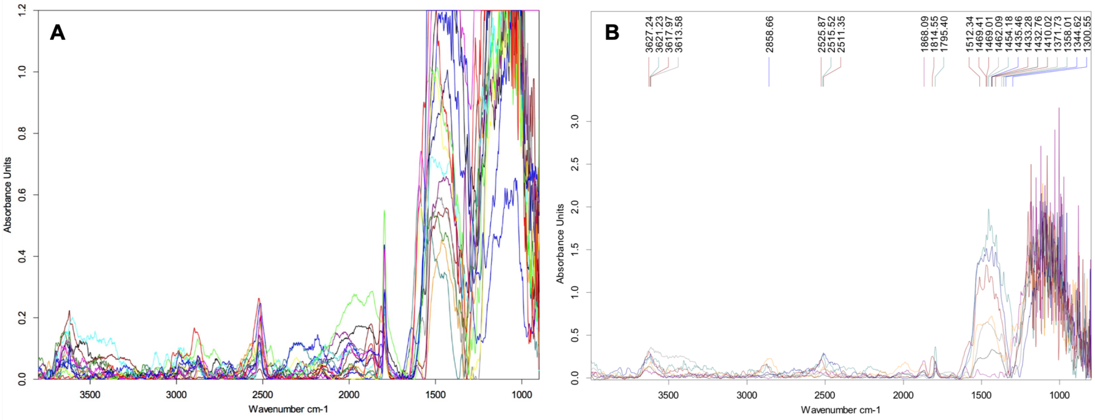

Figure S8. (A) Set of spectra collected from the light-coloured powder sample. The absence of any remarkable set of peaks in the ”organic” region of the spectrum, situated between 1500 - 1800 cm^{-1} (carbonyl signatures appear between 1630-1800 cm^{-1})
, is in clear contrast relative to the same region identified in the spectral collection for the dark-coloured powder shown in Fig. S7. Each point in the mapped region of the light-coloured powder sample (not shown) is represented by a different colour. (B) Set of spectra representative of a line in the mapped region of the light-coloured powder sample.

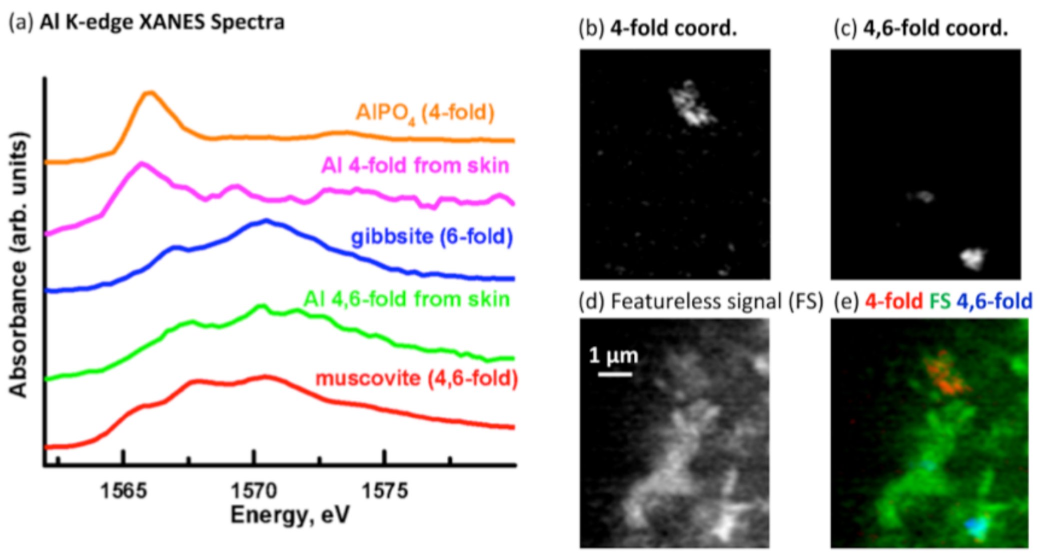

Figure S9. Aluminum component maps derived from the linear regression fitting of an Al K-edge image sequence using reference spectra. (a) Comparison of Al 4-fold coordinated and Al 4, 6-fold coordinated spectra derived by threshold masking of the component maps to the muscovite (4, 6-fold coordinated), gibbsite (6-fold coordinated Al) and AlPO4 (4-fold coordinated Al) reference spectra. Component maps (b-e). (b) 4-fold coordinated Al, (c) 4, 6-fold coordinated Al and (d) slow varying featureless signal (FS). (e) Color composite of the component maps (4-fold coordinated Al = red, featureless signal = green and 4, 6-fold coordinated Al = blue).

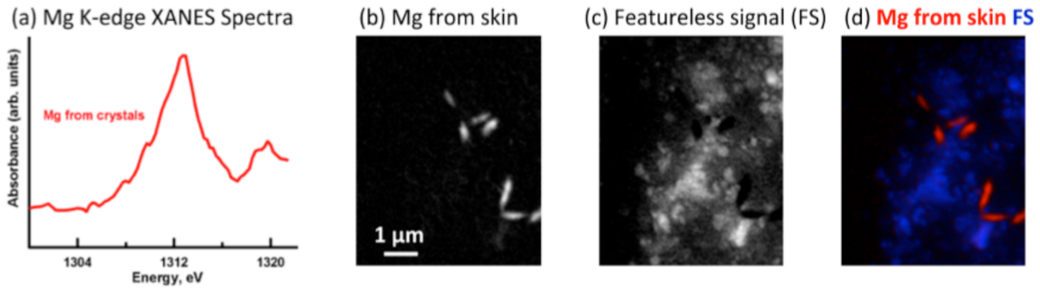

Figure S10. Magnesium component map derived from the linear regression fitting of an Mg K-edge image sequence using a spectrum taken from the image sequence. (a) Mg spectrum from the crystals (sediment). Component maps: (b) Mg and, (c) slow varying featureless signal (FS). Color composite of the component maps (Mg = red; featureless signal = blue).

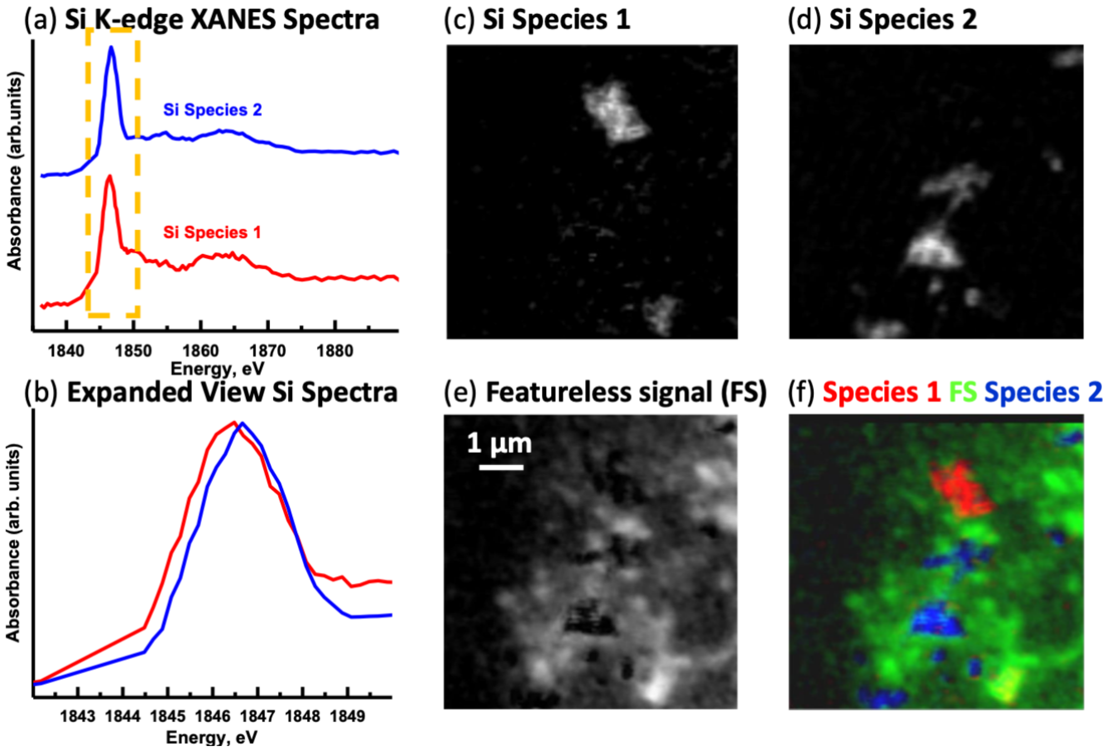

Figure S11. Silicon component maps derived from the linear regression fitting of an Si K-edge image sequence using spectra taken from the image sequence. (a) Si spectra (Si species 1 and Si species 2) derived by threshold masking of the respective component maps. (b) Overlay of the Si spectra. Orange box shows the area expanded from (a). Component maps: (c) Si Species 1, (d) Si Species 2 and (e) slow varying featureless signal (FS). (f) Color composite of the component maps (Si species 1 = red, featureless signal = green and Si species 2 = blue).
